# Diversification of terpenoid emissions proposes a geographic structure based on climate and pathogen composition in Japanese cedar

**DOI:** 10.1038/s41598-021-87810-x

**Published:** 2021-04-15

**Authors:** Tsutom Hiura, Hayate Yoshioka, Sou N. Matsunaga, Takuya Saito, Tetsuo I. Kohyama, Norihisa Kusumoto, Kentaro Uchiyama, Yoshihisa Suyama, Yoshihiko Tsumura

**Affiliations:** 1 Department of Ecosystem Studies, Graduate School of Agricultural and Life Sciences, The University of Tokyo, Tokyo, 113-8657 Japan; 2 Graduate School of Environmental Earth Science, Hokkaido University, Sapporo, 060-0809 Japan; 3 R&D Center, Green Technology System Division, Taikisha Ltd, Aiko-Gun, 243-0308 Japan; 4 National Institute for Environmental Studies, Tsukuba, 305-8506 Japan; 5 Forestry and Forest Products Research Institute, Forest Research and Management Organization, Tsukuba, 305-8687 Japan; 6 Field Science Center, Graduate School of Agricultural Science, Tohoku University, Osaki, 989-6711 Japan; 7 Graduate School of Life and Environmental Sciences, University of Tsukuba, Tsukuba, 305-8577 Japan

**Keywords:** Atmospheric chemistry, Biogeography, Evolutionary ecology, Forest ecology

## Abstract

Biogenic volatile organic compounds emitted from plants are important constituents of atmospheric chemistry and play a major role in the resistance of plants against various environmental stresses. However, little is known about how abiotic and biotic environments on a geographic scale relate to diversifications of the emission. Here, we present variations of terpenes stored in and emitted from leaves of a single species in a common garden, using genetically differentiated local populations of Japanese cedar, the most dominant and widely distributed tree species in Japan. Furthermore, we determined the composition of fungal communities in 50 locations, based on the presence or absence of 158 fungal species inhabiting the cedar. The results showed that terpenoids, especially those that are emitted, were highly diversified and geographically structured among the 12 populations. The total amount of stored terpenes was negatively affected by warm and less-snow climates. On the other hand, variations in some emitted terpenoid species among the populations were correlated to antagonistic fungal species inhabiting the Japanese cedar. We propose that the diversification of composition and amount of stored and emitted terpenoids in the tree species is not only structured by climate, but also antagonistic fungal communities through biological interactions.

## Introduction

Terrestrial ecosystems are responsible for the emission of large amounts of biogenic volatile organic compounds (BVOCs) into the atmosphere. BVOCs play an important role in atmospheric chemical processes^1,2^ as well as climatic processes^3,4^. Furthermore, BVOCs also have various ecological roles in response to abiotic stresses, such as heat, and biotic stresses, such as defense against insect herbivores, microbes, and fungi^5,6^. And BVOCs act as a communication tool among plants when they face biotic stresses^7,8^, and also mediate mutualistic interactions between microbes and plant against the stresses^9^. However, little is known about how climate and biotic environments such as species composition of pathogenic fungi on a geographic scale relate to diversifications of BVOCs.

Ecological interactions among organisms and environments, including plant–pollinator, plant–herbivore, and predator–prey interactions, evolve on a local scale^10–12^. Studies of geographical variation in ecologically interacting species should shed light on the question of how coevolution proceeds. In order to elucidate adaptive strategies for plant defense against pathogens and herbivores through BVOC emissions, it will be necessary to consider intraspecific variations of BVOCs in heterogeneous environments. In the case of angiosperm species, a variety of organisms, including herbivores and pollinators, interact and co-evolve with local plant populations through plant BVOCs creating complex networks within the system^13,14^.

Studies of variations in plant traits and pathogen communities along geographical gradients will provide unique insights into understanding the role of pathogens and abiotic environments in the evolution of plant defenses. In some tree species, climate and forest conditions are considered to be the major determinants of fungal community structure inhabiting the woods^15–17^. Dispersal limitations also play an essential role in maintaining regional endemism and influencing local community assembly of fungi^18^. Therefore, differences in regional pathogen composition as well as the climate, must be taken into account when dealing with interactions between trees and pathogens through BVOCs.

Geographical variations in the composition and amount of terpenoids stored in leaves have been studied in some gymnosperms, which were associated with insect or pathogen attack or demographic processes^19–21^. Unlike the stored terpenoids, however, there are few such studies on intraspecific geographical variations in terpenoids emitted as BVOCs. Thus, the mechanisms underlying BVOC production by plants in response to pathogen infection are not well known. It remains challenging to detect complex BVOC composition associated with ecological variation across multiple populations. In the present study, we hypothesized that BVOCs from gymnosperms are sensitive to the selection imposed mainly by the local assemblage of antagonistic fungi.

Japanese cedar, *Cryptomeria japonica* (Cupressaceae), is the most abundant gymnosperm tree species in Japan^22^, covering 20% of the forest area from the southern-most Okinawa island to the northern-most Hokkaido island when plantations are included^23^. We conducted an experiment to elucidate the geographical variations of BVOCs in *C. japonica* in a common garden with plants collected across the entire natural distributional range, and analyzed their relationship to variations in fungal communities and climate. Bridging evolutionary biology, ecology, and atmospheric chemistry, our study elucidates how BVOC emissions are geographically structured by climate conditions and antagonistic fungi using a genetically differentiated tree species with a large-biomass.

## Results

### Variations of terpenoids among cedar populations

**Figure 1:**
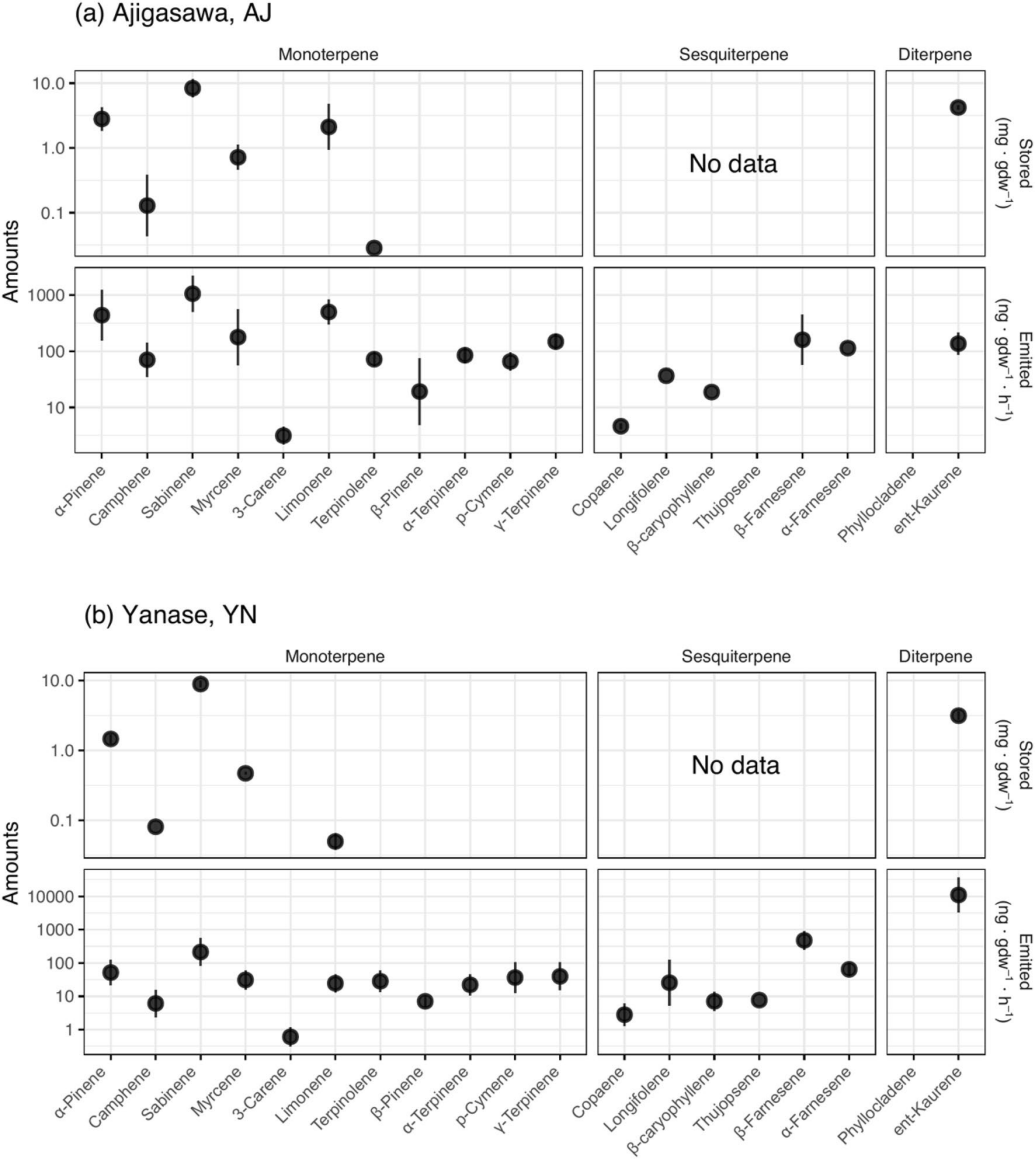
Stored and emitted terpenes detected in the case of Ajigasawa (above) and Yanase (below) population. Note that stored SQTs were not measured, and Sclarene was detected but not included in the analysis owing to the lack of an authentic standard.

**Figure 2:**
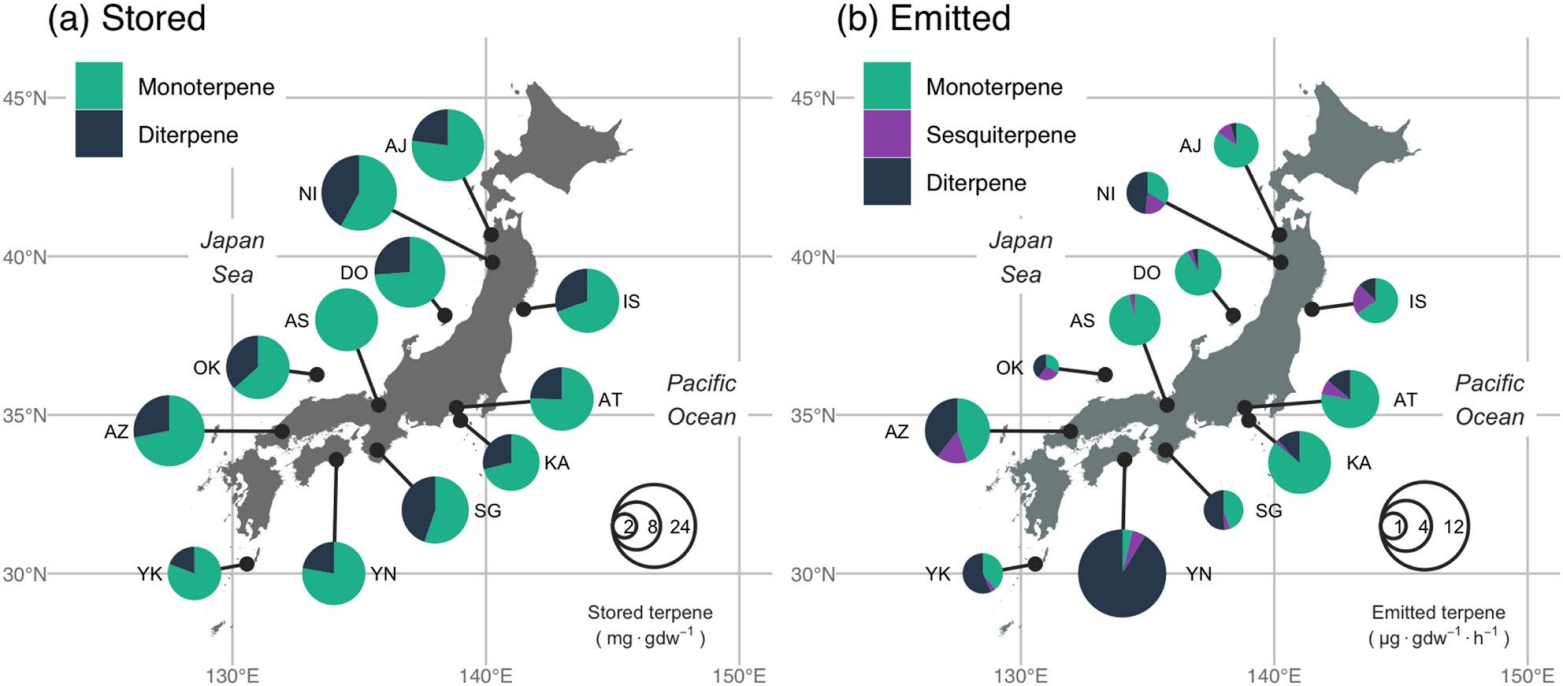
Stored (**a**) and emitted (**b**) terpenoids in *C. japonica* leaves grown in a common garden. The map was generated using GeoPandas (version 0.8.0; https://geopandas.org) and matplotlib (version 3.3.4; https://matplotlib.org) packages in Python 3.8.6. The source shape file was from the GADM database (version 3.6; https://gadm.org).

**Figure 3:**
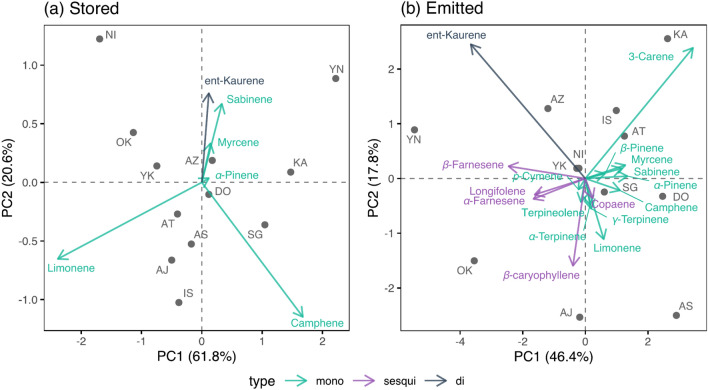
Principal component analysis of stored (**a**) and emitted (**b**) terpenes for each population. Populations at Pacific Ocean side, southwest populations, and Japan Sea side were segragated for emitted terpenes.

There were 11 kinds of monoterpenes (MTs), 6 kinds of sesquiterpenes (SQTs), and 2 kinds of diterpenes (DTs) stored in and emitted from *C. japonica* (Fig. 1). Although sclarene, a kind of DT, was also detected, it was excluded from the analysis owing to the lack of an authentic standard. The amount of total stored MTs and DTs were similar in the 6 Japan Sea side populations to the 6 Pacific Ocean side populations. Furthermore, the MTs/DTs ratio was not different among populations except for the AS population where no DTs except sclarene were detected (Fig. 2a). A principal component analysis of stored terpenoid compositions resulted in 82.4% of the overall variation being explained by the first two principal components, but their geographic structure was not clear (Fig. 3a).

The geometric mean of the emission rates of MTs, SQTs, and DTs among the populations ranged from 320.6–5299.0, 90.7–1024.1, and 26.9–11,038.9 (ng/gdw/h), respectively. The compositions and emission rates varied widely among populations, especially DTs, whose emissions varied strongly and were extremely high in the three southwest populations (YN, AZ, YK) (Fig. 2b). The first two principal components (PCs) explained 64.2% of the overall variation in the composition of emitted terpenes, and the geographical location of each population was segregated to three parts (Fig. 3b). A major section of the populations at the Pacific Ocean side (KA, IS, AT, SG) were mainly scored at first quadrant and high emission of 3-Carene, southwest populations (YN, AZ, YK) were scored at second quadrant and high emission of ent-kaurene and β-farnesene, and populations at the Japan Sea side (OK, AS, DO, AJ, NI) were mainly scored at third and fourth quadrants and high emission of β-caryophyllene and limonene.

### Correlation between terpenoids and climate, and fungal composition

The total amount of stored MTs and DTs were negatively correlated with the PC1 of climatic variables (warm and less snow environment, *P* = 0.016). The compositional ratio of each stored terpene was significantly affected by the total amount of stored terpene (MANOVA, *P* = 0.001) and climate PC1 (*P* = 0.025), and the climate PC1 was negatively correlated with limonene (*SI Appendix,* Fig. S3a). On the other hand, the total amounts of emitted terpenes did not correlate with the climate index (PC1 and PC2, both *P* > 0.1). The compositional ratio of each emitted terpene was significantly affected by the total amount of emitted terpenes (MANOVA, *P* = 0.008), climate PC1 (*P* = 0.050), and PC2 (*P* = 0.038), and climatic PCs positively correlated with ent-kaurene and negatively correlated with β-farnesene (*SI Appendix,* Fig. S3b).

**Figure 4:**
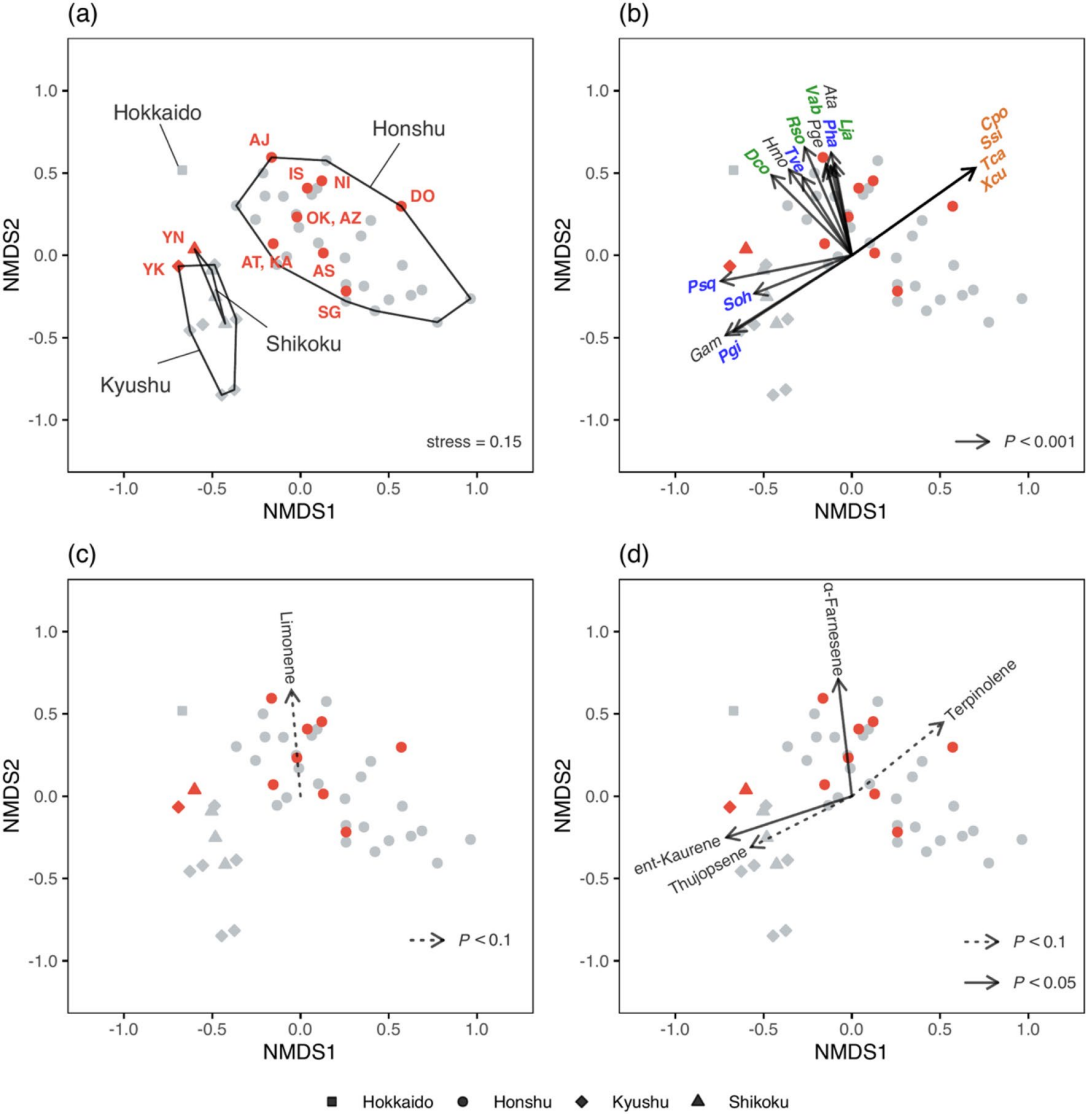
NMDS ordination by fungal composition of each population (red) and prefecture (grey) (k = 3, stress = 0.15). The four main islands are indicated by different symbols (**a**). There are 32 antagonistic species for *C. japonica,* which contribute significantly to the characterization among the local populations, but only 17 species are shown for simplicity (**b**). Note that leaf attacking fungi (green: Lja, Vab, Rso, Dco) characterized populations which were scored with low NMDS1 and high NMDS2, cellulolytic basidiomycetes (brown rot fungi, brown: Cpo, Ssi, Tca, Xcu) characterized populations which were scored with high NMDS1 and NMDS2, and lignocellulolytic basidiomycetes (white-rot fungi, blue: Pso, Pgi, Pha, Psq, Soh, Tve) characterized populations which were scored with low NMDS1, respectively. Abbreviations for fungal species and grouping were listed in Table S4. Stored Limonene (**c**) and emitted ent-kaurene, α-farnesene, thujopsene, and terpineolene (**d**) contributed significantly to the characterization among the local populations.

The fungal composition of each prefecture was discriminated by the locations of the four main islands in Japan (Hokkaido, Honshu, Shikoku, and Kyushu; Fig. 4a). Of the 158 fungal species, 32 antagonistic species for *C. japonica* showed a significant correlation with the NMDS coordinates (for 17 species, *P* < 0.001, Fig. 4b; for 15 species, *P* < 0.005, Table S1). Twenty species including leaf attacking fungi, *Diaporthe conorum, Leptosphaerulina japonica, Rhizoctonia solani, Valsa abietis, Guignardia sawadae, Cylindrocarpon* sp. were scored as having low NMDS1 and high NMDS2 values, and were significantly corresponded to emitted α-farnesene (*P* < 0.05) and marginally corresponded to stored limonene (*P* < 0.1)(Fig. 4b–d). Four species including brown rot fungi, *Postia caesius, Spongiporus sinuosa, Tremella candida,* and *Xeromphalina curtipes,* had high NMDS1 and NMDS2 values, and were marginally corresponded to emitted terpineolene (*P* < 0.1) (Fig. 4b,d). In addition, 8 species including white-rot fungi, *Phellinus gilvus, P. hartigii, Polyporus squamosus, Strobilurus ohshimae,* and *Trametes versicolor,* scored low NMDS1 and NMDS2 values, and were significantly corresponded to emitted ent-kaurene (*P* < 0.05) and marginally corresponded to thujopsene (*P* < 0.1)(Fig. 4b,d).

## Discussion

Stored limonene and emitted ent-kaurene and β-farnesene had climate clines (e.g. warm and less snow), but other terpenoids did not have obvious climate clines (*SI Appendix,* Fig. S3). These suggest that both climate clines and other environmental factors such as biotic pressure may regulate these adaptive evolutions^21^. Our analysis of fungal community structure, although qualitative suggested that antagonistic fungi inhabiting *C. japonica* were one of the key drivers of the evolution of defensive chemicals such as terpenoids. Several studies have demonstrated that conifers have functionally diverse terpene synthase genes that produce a wide variety of terpene products, and that these genes have evolved through gene duplications and minor sequence divergences^5^. These minor changes in the active center structure can lead to major changes in product profiles, and the presence of abundant terpenoid genetic diversity has resulted in rapid evolutionary adaptations to novel biotic interactions, and to new abiotic stresses in plant habitats^5,24^. Neutral gene analysis suggests that some populations such as Yakushima, Oki, and Shingu are thought to be refugia in the last glacial period, forming a geographic genetic structure^25^, which is not entirely consistent with the geographic pattern of BVOC emissions identified in this study. This partial discrepancy between the geographic structure of BVOC emission and that of neutral genes may indicate that adaptive genes have been selected more rapidly. The geographically structured diversification of BVOC emissions might be an adaptive rather than a random model of evolution. This finding indicates that the different strategies of BVOC emission would be favored under different environments together with other carbon allocations, such as growth^26^ and root exudation^27^. The defense-growth trade-off might be a driver of the geographic variation of traits^28^ in *C. japonica*, but diversification of strategies for water and nutrient utilization should be another driver in heterogeneous geology and climate where water and nutrient availabilities are much different among the locations of the populations^27^. During the last glacial period, the distributions of fungi inhabiting Japanese cedar may have been geographically structured, although they would have also been inhabiting other hosts. Then, the evolution of BVOC emissions at the local scale may have occurred during the segregation and migration from the refugia.

*Diaporthe conorum, Leptosphaerulina japonica, Rhizoctonia solani, Valsa abietis, Guignardia sawadae,* and *Cylindrocarpon sp.* are leaf or fruit attacking fungi^26,29–31^. *Corticium portentosum, Spongiporus sinuosa, Tremella candida,* and *Xeromphalina curtipes* are cellulolytic basidiomycetes (brown rot fungi^32^), and *Phellinus gilvus, Strobilurus ohshimae,* and *Polyporus squamosus* are lignocellulolytic basidiomycetes (white-rot fungi^33–36^). Surprisingly, the fungal compositions in the northern populations were characterized mainly by the pathogens that attack the leaves and fruits (Fig. 4). On the other hand, the composition of populations which were located at the Japan Sea Side was characterized mainly by the brown-rot fungi, and the white-rot fungi, which were distributed in the all populations without deviation (Fig. 4). Temperature is thought to be one of the candidate drivers that influence fungal diversity^15^. For example, the presence of brown rot species inhabiting *C. japonica, Xeromphalina spp.* correlated with higher mean annual temperature^16^. On the other hand, dispersal limitations of fungal species also play a significant role in shaping local community assembly^18^. These biases in the functional groups of pathogens in each local population may be responsible for the evolution of interactions with the host cedars and the emergence of features of BVOC emissions.

The functional role of each terpenoid on pathogens can disentangle the relationships between the composition of BVOCs emitted from the tree species and the community structure of the microbiome at a local scale. There is some evidence that some kinds of BVOCs act as a defensive material against pathogens and pests. Since oxygenated monoterpenoids have antimicrobial properties, it is also necessary to focus on the oxidation products derived from BVOCs as well. In fact, elemol, the thermal oxidation product of hydycaryol in cedar leaves, showed high activity against the wood decay fungi^37^. Kaurene, known to have antibacterial effects after oxidation^38^, may interact with some pathogens. Thujopsene also acts as an antifungal VOC, and reduces mycelial growth after oxidation^39^. D-limonene synthase downregulation induced resistance against fungus through enhanced accumulation of monoterpene alcohols and activation of defense responses in plants^40,41^.

In this study, DTs were emitted in large amounts from the southwestern populations (Fig. 2b). DTs have a high molecular weight, and in the case of artificial cultivars, larger quantities are emitted from the southern varieties than those from central Japan^42^. When exposed to air, DTs contribute to the trapping and encrusting of insects due to their high viscosity and polymerization, and they can be toxic to pathogens and insects^6^. If the bidirectional BVOC flux is greater than previously thought^43^, and the deposition on the surface of the leaf is greater, it may be effective against pathogens that attempt to invade the inside of a leaf from the leaf surface. Kaurene emitted in large quantities from the southwestern populations may be act as a defense against other microbes. On the other hand, a study on a shrub species that emitted kaurene did not originate from de novo biosynthesis and was emitted in a temperature-dependent manner^44^. We cannot fully rule out the possibility that the climatic conditions in the common garden may alter the basal emission rates from those expected in the original locations. It is also possible that some reaction norms of BVOCs emissions for each population may differ to the biotic environments (e.g., fungal composition) in the common garden. The geographically structured variation of BVOCs emissions into the atmosphere will probably be more noteworthy in the future.

The emission rates of MTs, SQTs, and DTs from *C. japonica* measured in this study using saplings were significantly higher than those measured in a previous study using canopy trees of *C. japonica*^42^. This difference might be due to the individual tree size, given that the rate of MTs emitted from *C. japonica* saplings^45^ was similar to the rate measured in this study. Moreover, if smaller individuals invest more in defense against the pathogen than larger individuals, they may be more effective in avoiding death due to the diseases.

Diversification and magnitude of BVOC emissions, including MTs, SQTs, and DTs, which are known to be major BVOC constituents in local populations, may affect on atmospheric chemistry and physics^2,4^ at local and regional scales. If tree-pathogen interactions determine the direction of terpene emission, it indicates that the evolution caused by the interactions between organisms may also affect atmospheric chemical processes. BVOC sources can also lead to the evolution of plant chemical diversity, creating volatile blends that help to identify the fungal as well as insect species^46,47^. Our knowledge of BVOC emission under multiple attacks is still limited to a conclusive explanation about how plants determine BVOC induction patterns^46^. However, the results of our present study may shed light on such patterns. Further research on the mechanisms behind our observation is warranted.

## Conclusions

The composition and amount of BVOCs emitted as a defensive material might differ significantly on a local scale owing to the evolutionary consequences of ecological interactions among host trees, climate and pathogens. Since the functional traits of a dominant tree species usually has cascading effects on the ecosystem, such geographical diversification of BVOCs found in a cedar with the largest biomass in Japan may affect not only on biotic interactions but also even atmospheric chemical processes. Furthermore, we not only have to pay attention to the biotic interactions in various artificial cultivars of Japanese cedar that have been planted on a large scale nationwide since the 1950’s, but also breeding that considers the interactions between pathogens and herbivorous insects via BVOC that are required in the future.

## Materials and methods

### Common garden and materials

The common garden was located at the Field Science Center, Tohoku University (38.78° N, 140.73° E, 250 m a.s.l.) on the Tertiary tuff with volcanic ash. The mean annual temperature was 10.2 °C, and the mean annual precipitation was 1650 mm^48^. We conducted field measurements and sampling from the 1st to 17th of July 2019 for terpenes, and the mean temperature, total precipitation, in this period were 19.0 °C and 3.1 mm, respectively. We chose three cedar saplings planted in the common garden in 2016 from each of the 12 populations throughout the entire natural geographical range of *C. japonica* (*SI Appendix,* Table S2). We got the permissions for collection of the plants from the owners. The distance between the trees was 2 m, and the tree height ranged from 1 to 3 m, and every tree received almost full sunlight.

Natural *C. japonica* populations are distributed widely in Japan from Yakushima Island to northern Honshu (YK and AJ in this study). The geographical variation between natural forests of *C. japonica* has been investigated, focusing on growth^33^, clonal reproduction^49^, and utilization of calcium and water^34^, however, interactions between functional traits of cedar and pathogen were not clearly investigated. Differences in stored diterpene components in leaves were also found between the populations on the Japan Sea side and the populations on the Pacific Ocean side^50^. Populations on the Pacific Ocean side of Japan are clearly different in genetics from those on the Japan Sea side^51^, and the historical process of genetic divergence in *C. japonica* after the last glacial maximum (21,000 yeas BP) was proposed and there was evidence of multiple refugia, such as high genetic diversity in marginal populations^36,51^.

### Collection of volatile and stored terpeniod samples

Foliar emission gas was collected using the dynamic branch enclosure technique. Intact branches were enclosed in a fluorinated ethylene propylene (FEP) bag with a purge air inlet that supplies VOC-scrubbed compressed air (*SI Appendix,* Fig. S1). Two enclosure air samples (2 L each) were collected simultaneously for MTs and SQTs/DTs measurements in adsorbent tubes. After VOC sampling, the branch in the enclosure was collected for stored terpenoid measurements. Details of the sampling methodology are described in SI Appendix.

### Chemical analysis

Adsorbent tube samples for MTs and SQTs/DTs were analyzed by thermal desorption gas chromatography coupled with a mass selective detector and a flame ionization detector (TD-GC/MSD/FID) and sorbent extraction followed by GC/FID, respectively. Stored terpenoids in collected branches were extracted with solvent and measured using a GC/MS. Details of the chemical analysis including the GC operation protocols, detection limits, and uncertainties are provided in SI Appendix.

All methods complied with relevant institutional guidelines.

### Data analysis

To explore whether diversification in terpenoid compositions was due to adaptive responses to climate and inhabiting fungi heterogeneity across the cedar populations, we investigated the across-population correlations between terpenoids that showed signs of diversifying selection and the climate indices, and the composition of fungi.

Average climate data at the original location of each cedar population were obtained for the period 1980–2010 from the nearest weather stations (http://www.data.jma.go.jp/obd/stats/etrn/index.php). The following climate variables were used: annual mean temperature, annual precipitation, global horizontal irradiance, sunshine duration and maximum snow depth. To minimize type I error in the correlation analysis, the five climatic variables were summarized into main components by means of a PCA using R^52^. The first two PCs contributed to 91.3% of the overall variance. PC1, which contributed to 74.9% of the overall variance, was positively correlated with temperature and solar energy, and negatively correlated with maximum snow depth, suggesting a warm and mild environment. The sampling sites located on the Japan-Sea side had a low PC1 score. PC2, which contributed to 16.4% of the total variance, was positively correlated with precipitation (*SI Appendix,* Fig. S2).

To investigate the relationship between variations in climatic factors and terpenoid compositions, we fitted a multivariate linear model with the isometric logratio transformed^53^ terpenoid composition as response variables. Rare terpenes, which were below the detection limit in more than half of the specimens, were excluded from the analysis. The total amount of terpenoids in each sample and the PC1 and PC2 scores for the climatic factors were included in the model as explanatory variables. We performed a MNOVA to test the significance of the explanatory variables. Singular value decomposition was performed on inverse isometric logratio transformed matrices of the coefficients of the explanatory variables estimated by the multivariate linear model to show the effect of each explanatory variable on terpene composition^54^. Data of stored and emitted terpenoids in 12 populations were shown in *SI Appendix,* Tables S3, S4.

The fungal species composition of each prefecture was analyzed based on previous data^55^. So far, 208 fungal species using Japanese cedar as a host have been identified in Japan, of which the distribution of 50 species is unknown^55^. We therefore analyzed the species composition of the fungal community for the remaining158 species based on the data. Due to the lack of numbers of fungal species reported on remote islands such as Oki island, Yakushima, and Sado (Donden), we used data on fungal composition from closest locations in the mainland (Shimane for the OK population; Kagoshima for the YK population; Niigata for the DO population).

We performed a non-metric multidimensional scaling (NMDS) based on Jaccard distances calculated from the presence/absence data of fungal species, to examine geographic trends in fungal composition reported in 46 prefectures in Japan, excluding Okinawa (*SI Appendix,* Fig. S4). We performed vector fitting to investigate the relationship between the NMDS coordinates constructed from the fungal community data and the terpenoid composition^56^.

## Supplementary Information

Supplementary Information.

## Data Availability

All data are available in Supplemental information. Additional data that support the findings of this study are available from the corresponding author upon request.
