# Supplementary material for: Diversification of terpenoid emissions proposes a geographic structure based on climate and pathogen composition in Japanese cedar
Source: Sci Rep. 2021 Apr 15;11:8307. doi: 10.1038/s41598-021-87810-x (PMC8050256; doi:10.1038/s41598-021-87810-x)
Supplement: Supplementary file 1 — Supplementary Information. [file 41598_2021_87810_MOESM1_ESM.docx]

Supplementary Information for

Diversification of terpenoid emissions is geographically structured by climate and pathogen composition in Japanese cedar.

Tsutom Hiura^1*^, Hayate Yoshioka^2^, Sou N. Matsunaga^3^, Takuya Saito^4^, Tetsuo I. Kohyama^2^, Norihisa Kusumoto^5^, Kentaro Uchiyama^5^, Yoshihisa Suyama^6^, and Yoshihiko Tsumura^7^

*Corresponding author: Tsutom Hiura

**Email:**  [hiura@g.ecc.u-tokyo.ac.jp](mailto:hiura@g.ecc.u-tokyo.ac.jp)

**This file includes:**

Supplementary text

Figures S1 to S4

Tables S1 to S4

SI References

Supplementary Information Text

**Detailed Experimental Procedure**

**Collection of volatile terpenoid samples.**

Foliar emission gas was collected using the dynamic branch enclosure technique (e.g., Ortega and Helmig, 2008). Intact branches were enclosed in a fluorinated ethylene propylene (FEP) bag (25 cm x 60 cm, GL Sciences Inc.) equipped with two sampling ports. Purge air was supplied from compressed air cylinders (Tomoe Shokai Co., LTD.) into the enclosure through an inline charcoal filter (GL Sciences Inc.). The flow rate was maintained at 5 L min^-1^ using a variable area flow meter with needle valve (KOFLOC RK1250). The air temperature was measured inside the enclosure using a Teflon-sheathed thermocouple. Enclosures were allowed to equilibrate for more than 12 h prior to sampling. The flow rate of the purified air was kept at 5 L min^-1^ during equilibration and sampling.

Enclosure air was sampled by drawing 200 mL min-1 of enclosure air through

an adsorbent tube for 10 min (sample volume: 2 L) using an air sampling pump (GSP-

300FT-2, GASTEC). Two types of adsorbent tubes were used for sample collection:

thermal desorption (TD) adsorbent tube (1/4” O.D. x 3.5” long) filled with 200 mg of

Tenax TA and 100 mg of Carbotrap B (SUPELCO, Bellefonte, USA) for monoterpene

measurements, and sorbent extraction (SE) adsorbent tube (6mm O.D. x 76mm long)

filled with HayeSep Q for sesqui-/di-terpenes measurements. After sample collection,

TD adsorbent tubes were sealed with a TDS3 storage container and kept at -18°C until

analysis.

SQTs and DTs in the enclosure bag were collected using approximately 60 mg

of an absorbent HayeSep Q (Hayes Separations In., Bandera TX, USA) in a 6 mm

O.D. and 76 mm length, whose end was tightened to make the liquid drop lead into a glass vial. The flow rate and sample collection time were 200 mL min-1 and 10 min,

respectively. The collected SQTs and DTs were extracted by approximately 1.5 mL of

hexane (special grade, Fuji Film Wako, Osaka, Japan) and dropped into a 2 mL glass

vial immediately after the sampling, and closed using a plastic cap with PTFE liner. The

extracts were stored at -18 ºC until analysis.

**Measurements of volatile monoterpenes.**

TD samples were analyzed using a custom-built thermal desorption unit/gas chromatograph/mass selective detector/flame ionization detector (TD-GC/MSD/FID, Agilent 6890/5973). The TD unit was modified based on a system that was used to analyze foliar gas samples (Saito and Yokouchi, 2008). TD samples were purged at 40 °C with 50 mL min^-1^ nitrogen for 1 min, then heated to 250 °C using a cartridge heater. The thermally desorbed analytes are transferred from TD tube in a flow of 10 mL min^-1^ helium to a focusing trap consisting of a 1/32-inch O.D. Sulfinert-treated stainless steel tube packed with solid adsorbent material (2 mg Tenax TA and 2 mg Carboxen 1000, Supelco), which was held at approximately -130 °C with a free piston stirling cooler. Subsequently, the focusing trap was flash-heated to 180 °C with a nichrome wire heater, and the analytes were backflushed with helium carrier gas at a flow of 1.5 mL min^-1^ to an HP-5 capillary column (60 m long, 0.32 mm I.D., 1 μm film thickness; Agilent) in a GC oven. The GC oven temperature was initially maintained at 30 °C for 2 min, then ramped up to 160 °C at 4 °C min^-1^, 45 °C min^-1^ to 300 °C, and finally held at 300 °C for 10 min. Following the chromatographic separation, the eluting analytes were split equally between the MSD and the FID via a two-way splitter with makeup gas (G3180B, Agilent) and two fused-silica capillary columns. Monoterpenes were identified and quantified by FID with liquid standards prepared from pure authentic standards dissolved in methanol. The MSD was operated in SCAN mode and used for further confirmation of the compounds. Linear calibration curves were obtained for monoterpenes over the entire range of expected concentrations.

**Measurements of volatile sesqui- and di-terpenes.**

SQTs and DTs in the sample extracts were separated and analyzed using a gas chromatography-mass spectrometer (GC-MS, Agilent 5973N MS equipped with Agilent 6890N GC, Agilent, Santa Clara, CA, USA). Approximately 10 ng of cyclopentadecane dissolved in hexane at concentration of around 10ng μL^-1^ was added into the sample extract as an internal standard. The extract was concentrated with a gentle, pure nitrogen flow to approximately 10-20 μL. The concentrated extract was injected into a split-splitless injector equipped on the GC. The injector was set to function in splitless mode and heated to 320 ºC. SQTs and DTs were separated using a capillary column Agilent HP-5, (60 m 0.25 mm I.D., film thickness 0.25 μm) with a helium carrier gas. The GC column oven temperature was programmed to be started at 60 ºC and ramped to 120 ºC at 30 ºC min^-1^, then ramped to 320 ºC at 2 ºC min^-1^ and kept at 320 ºC for 10 min. The ion source of the MS was kept at 230 ºC. The MS analysis was conducted in selected ion mode (SIM). SQTs and DTs were qualified by comparing their retention time with their authentic standards and were quantified based on the ratio of MS response between the target and the internal standard (known amount).

**Calculation of VOC emission rates.**

VOC emission rates E (ng gdw^-1^ h^-1^) were calculated based on equation (1) below.

$E=\frac{F_{in}\times(M-M_{blank})\times60}{F_{samp}\times W_{dry}\times t_{samp}}$ (1)

where, F_in_, F_samp_, M, M_blank_, W_dry_ and T_air_ represent purified air flow rate into the enclosure (L min^-1^), sampling flow rate (L min^-1^), target VOC mass (ng), target VOC mass in the blank sample (ng), sample leaf dry weight (gdw), and sampling time in the enclosure (min), respectively. The blank sample was collected from the same enclosure system without a plant branch at the sampling site under the same conditions as the intensive sampling.

**Calculation of the basal emission rate.**

The basal emission rate E_s_, which is modelled as the VOC emission rate at standard leaf temperature of 30 ºC (T_s_), was calculated based on G93 model (Guenther et al., 1993). T represents inside temperature of air monitored by a thermocouple set at a place to avoid direct sunlight in the bag (17.5 ^0^C < T < 32.2 ^0^C). The equation of the G93 model for monoterpenes is as follows: We applied equation (2) to calculate the E_s_ also for SQTs and DTs. Equation (2) can be transformed into (2)’. The coefficient β was basically obtained as a slope of x (T-T_s_) – y (ln E) scatter plot drawn with several 10 emission data collected over a wide temperature range, and the ln Es can be obtained as the y-axis intercept of the regression line (see equation (2)’). However, in this study, both the emission data and temperature range were quite limited because we focused on variation and number of individuals of Japanese cedar. Therefore, we applied the β of MTs, SQTs, and DTs for Japanese cedar reported in Matsunaga et al. (2011 and 2012), which are 0.17 (MTs), 0.20 (SQTs) and 0.21 (DTs).

$E=E_{s}\exp\left\{ \beta\left( T-T_{s} \right) \right\}$ (2)

$\ln E=\beta\left( T-T_{s} \right)+\ln E_{s}$ (2)’

**Measurements of stored isoprenoids.**

The branch in the enclosure was cut at the end of the enclosure bag after VOC sampling and wrapped with wet absorbent cotton and aluminum foil to avoid desiccation and VOC evaporation. The branch was stored at -18 ºC until further sample preparation.

Plant isoprenoids contained in the needle leaves were extracted and were measured using a GC-MS (SHIMADZU GCMS QP-2010 ultra). The detailed procedure is as follows. Frozen needle leaves were cut into be several mm of pieces and crushed using a stainless mill (MX-1100XTM, WARING COMMERCIAL, Stamford, CT, USA) for several 10 seconds. The crushed leaf powder was removed to a polyethylene bag and stored at -18 ºC until solvent extraction. A small portion of the powder was removed to weigh in a cuvette and dried at 105 ºC for 48 h to obtain the weight of the water content. One gram of the rest of the powder was steeped in 20 mL of hexane containing approximately 0.05 mg mL^-1^ of cyclopentadecane as an internal standard and was kept at room temperature for 24 h. 1 μL of the supernatant from the extract was injected into a GC-MS equipped with a split-splitless injector heated at 240 ºC. Isoprenoids were separated using a GC capillary column (DB-5ms 30m, 0.25 mm I.D., film thickness 0.25µm) with helium carrier gas. The GC column oven was programmed to start at 40 ºC and raised to 280 ºC (4 ºC min^-1^) and kept for 7 min. The isoprenoids were qualified and quantified by the MS in scan mode. Its ion source was maintained at 200 ºC. Quantification was based on the ratio of MS response intensity between the target compound and the internal standard. Only MTs and DTs were quantified because SQTs separation was not appropriate for quantification.

SUPPLEMENTARYFIGURES:

**Figure S1.**

Measurement system of BVOCs emitted from *C. japonica* leaves in a common garden.


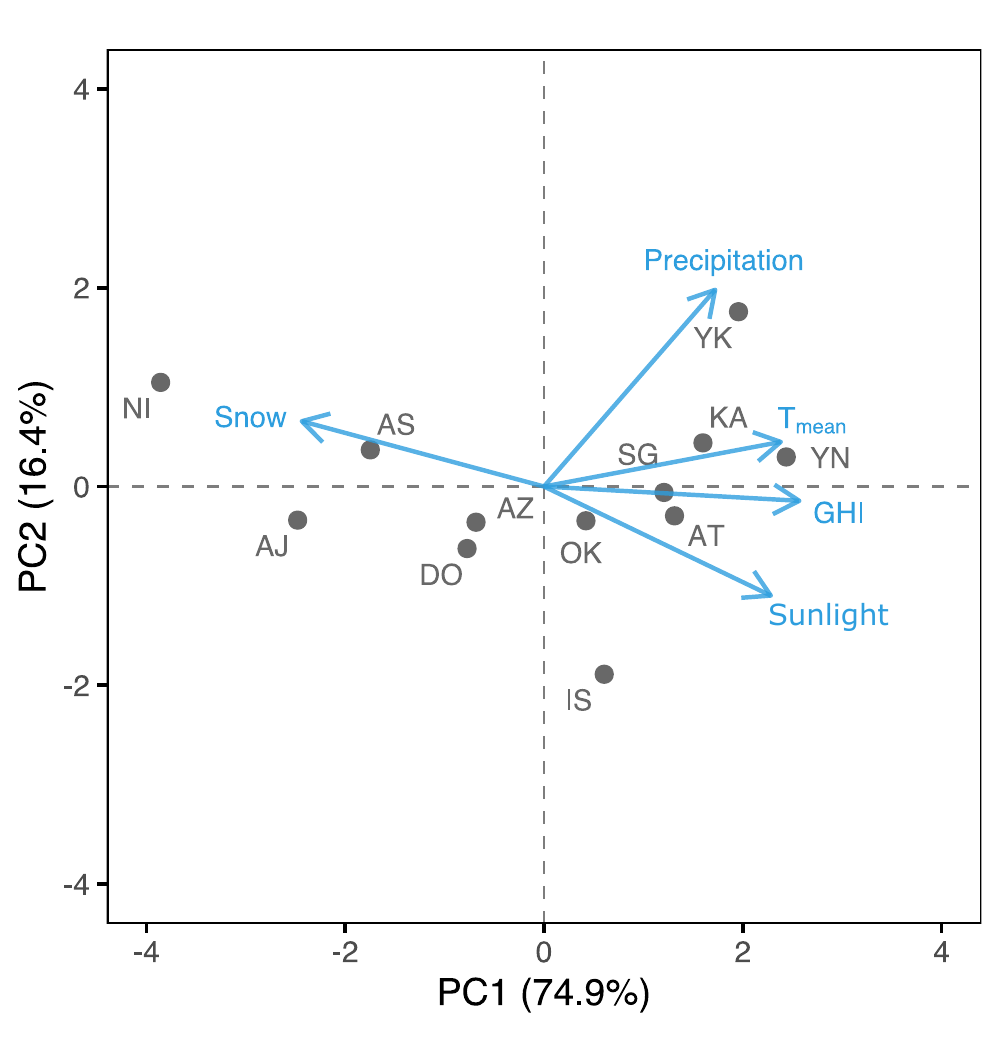


**Figure S2.**

Principal component analysis of five climate factors and populations. Precipitation, Snow, T_mean_, GHI, and Sunlight indicate annual precipitation (mm), maximum snow depth (cm), annual mean temperature (^0^C), global horizontal irradiance (kwh/m^2^), sunlight time (h), respectively. PC1 explained 74.9 % of the variance, was positively correlated with temperatures and solar energy, and was negatively correlated with maximum snow depth, thus suggesting a warm and mild environment. PC2 explained 16.4 % of variance and was positively correlated with precipitation.


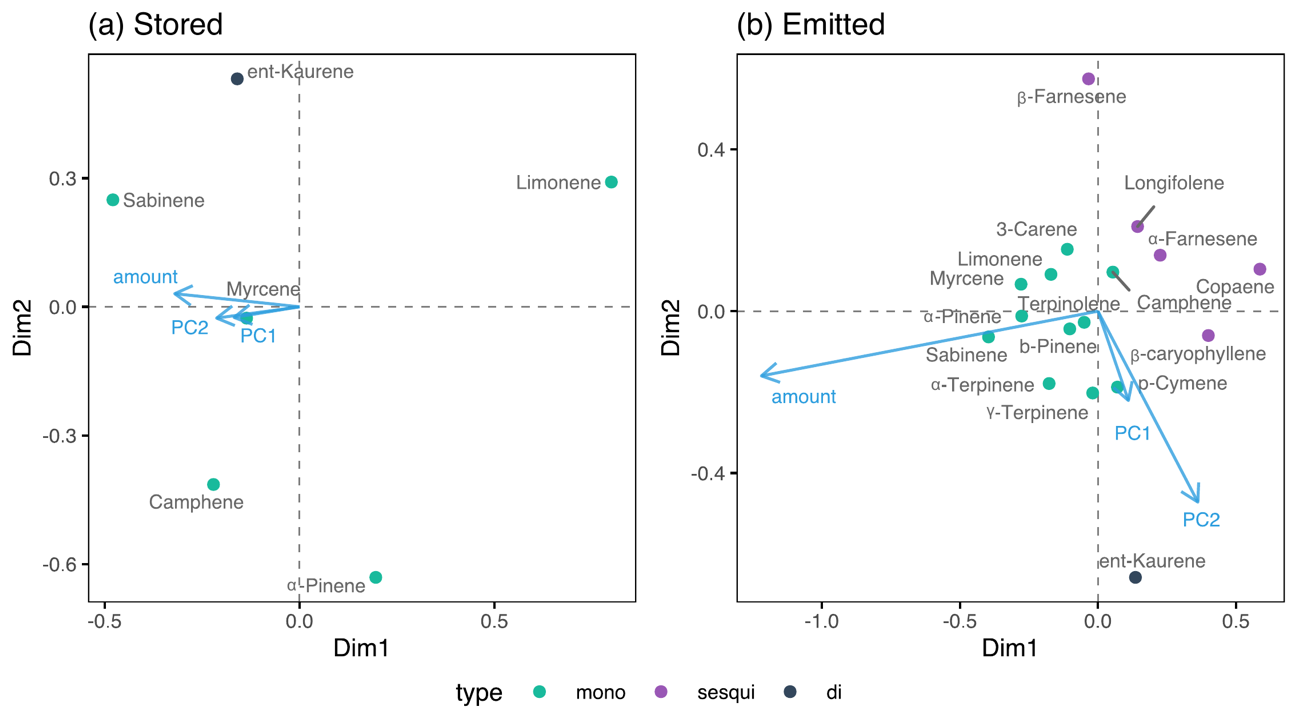


**Figure S3.**

Biplot of a multivariate linear model of climate factors (PC1 and PC2 in Fig.S3) and the total amount of stored (a) and emitted (b) on each terpene. The total amount (P = 0.001) and PC1 (P = 0.025) had significant effects on the compositional rate of each stored terpene, especially negatively on limonene (a). The total amount (P = 0.008), PC1 (P = 0.050), and PC2 (P = 0.038) had significant effects on the compositional rate of each emitted terpene, especially negatively on β-farnesene and positively on ent-kaurene (b).


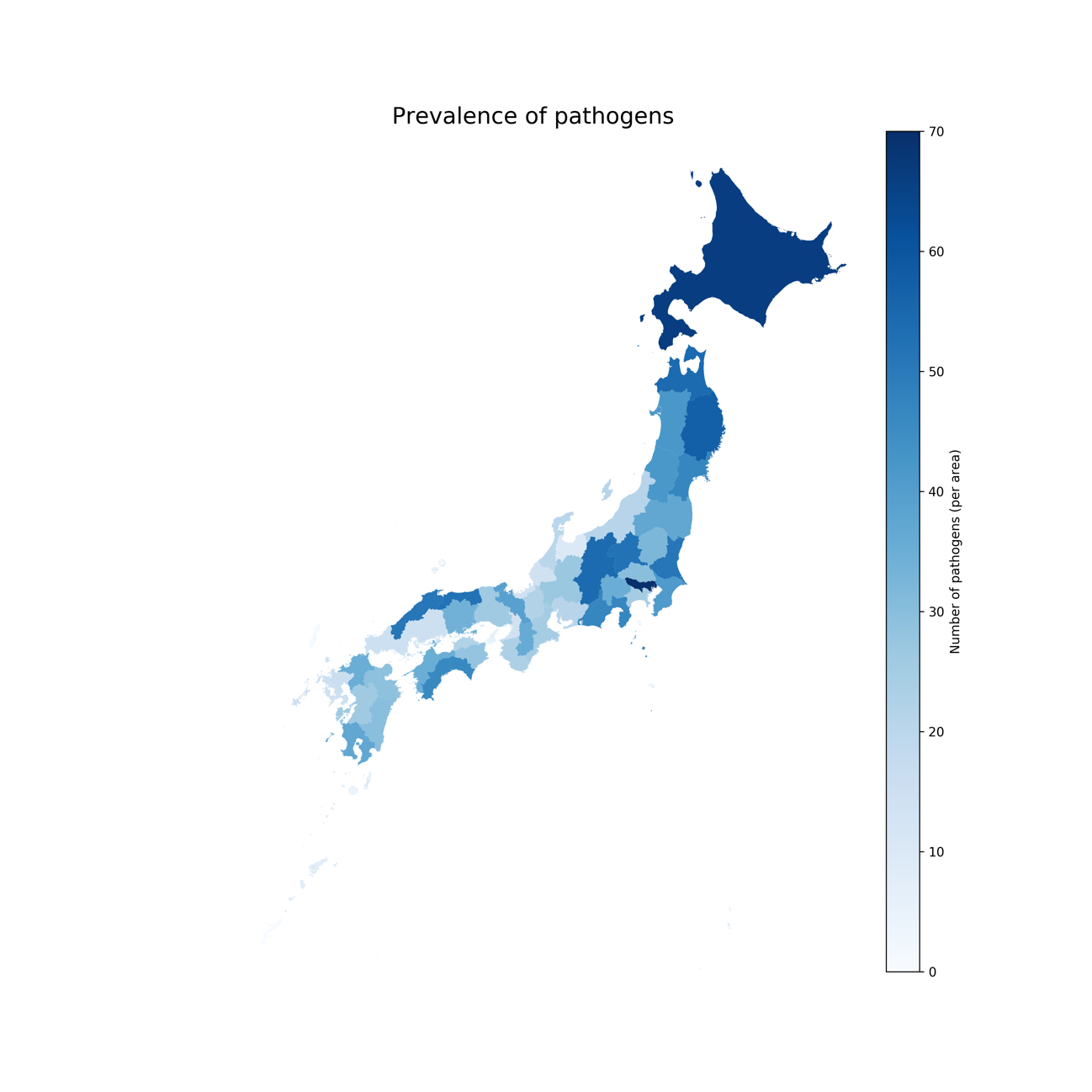


**Figure S4.**

Number of pathogens in each prefecture and island. Note that the distributional area of *C. japonica* in Hokkaido was eliminated in the Oshima peninsula, the southern part of Hokkaido. The map was generated using GeoPandas (version 0.8.0; <https://geopandas.org>) and matplotlib (version 3.3.4; <https://matplotlib.org>) packages in Python 3.8.6. The source shape file was from the GADM database (version 3.6; <https://gadm.org>).

**Table S2.**

Location of 12 *C.japonica* populations

| Population (abbreviation) | Latitude (N°) | Longitude (E°) | Elevation (m) |
| --- | --- | --- | --- |
| Ajigasawa (AJ) | 40.68 | 140.21 | 297 |
| Nibetsu (NI) | 39.81 | 140.26 | 366 |
| Donden (DO) | 38.14 | 138.38 | 790 |
| Ashu (AS) | 35.31 | 135.77 | 886 |
| Oki (OK) | 36.27 | 133.33 | 397 |
| Azouji (AZ) | 34.48 | 131.96 | 1060 |
| Ishinomaki (IS) | 38.33 | 141.49 | 159 |
| Ashitaka (AT) | 35.23 | 138.84 | 805 |
| Kawazu (KA) | 34.83 | 139.00 | 634 |
| Shingu (SG) | 33.89 | 135.71 | 583 |
| Yanase (YN) | 33.59 | 134.10 | 762 |
| Yakushima (YK) | 30.30 | 130.57 | 1047 |

**SI References**

1. Cheng SS, Lin HY, Chang ST (2005) Chemical composition and antifungal activity of essential oils from different tissues of Japanese cedar. *J Agrc Food Chem* 53: 614-619.
2. Fukui Y, Miyamoto T, Tamai Y, Yajima T (2018) Use of DNA sequence data to identify wood-decay fungi likely associated with stem failure caused by windthrow in urban trees during a typhoon. *Trees* 32: 1147-1156.
3. Guenther AB, Zimmerman PR, Harley PC, Monson RK, Fall R (1993) Isoprene and monoterpene emission rate variability: model evaluations and sensitivity analyses. *J Geophys Res* 98 https://doi.org/10.1029/ 93jd00527
4. Hasegawa E, Ota Y, Hattori T, Sahashi N, Kikuchi T (2011) Ecology of *Armillaria*  species on conifers in Japan. *For Pathol* 41: 429-437.
5. Hirooka Y, Masuya H, Akiba M, Kubono T (2013) Sydowia japonica, a new name for *Leptosphaerulina japonica* based on morphological and molecular data. Mycol Progr 12: 173-183.
6. Homma et al. (2007) Lignin-degrading activity of edible mushroom *Strobilurus ohshimae* that forms fruiting bodies on buried sugi (*Cryptomeria japonica*) twigs. *J Wood Sci* 53: 80-84.
7. Matsunaga SN et al. (2011) Monoterpene and sesquiterpene emissions from Sugi (*Cryptomeria japonica*) based on a branch enclosure measurements. *Atmos Pollut Res* 2: 16–23.
8. Matsunaga SN et al. (2012) Determination and potential importance of diterpene (kaur-16-ene) emitted from dominant coniferous trees in Japan. *Chemosphere* 87: 886–893.
9. Nakamura H, Ikeda K, Arakawa M, Akahira T, Matsumoto N (2004) A comparative study of the violet root rot fungi, *Helicobasidium brebissonii* and *H. mompa*, from Japan. *Mycol Res* 108: 641-648.
10. Ortega J, Helmig D (2008) Approaches for quantifying reactive and low-volatility biogenic organic compound emissions by vegetation enclosure techniques – Part A. *Chemosphere* 72: 343-364.
11. Ota Y et al. (2014) Taxonomy and phylogenetic position of *Fomitiporia torreyae*, a causal agent of trunk rot on Sanbu-sugi, a cultivar of Japanese cedar in Japan. *Mycologia* 106: 66-76.
12. Rizzo DM, Rentmeester RM, Burdsall HH (1995) Sexuality and somatic incompatibility in *Phellinus gilvus*. *Mycologia* 87: 805-820.
13. Ruhl V, Lotz-Winter H, Neuss A, Piepenbring M, Zorn H, Ruhl M (2017) Comprehensive analysis of the volatilome of *Scytinostroma portentosum*. *Mycol Progr* 17: 417-424.
14. Saito T, Yokouchi Y (2008) Stable carbon isotope ratio of methyl chloride emitted from glasshouse-grown tropical plants and its implication for the global methyl chloride budget. *Geophys Res Lett* 35: L08807
